# The Protective Effects of Carrageenan Oligosaccharides on Intestinal Oxidative Stress Damage of Female *Drosophila melanogaster*

**DOI:** 10.3390/antiox10121996

**Published:** 2021-12-15

**Authors:** Kun Yang, Qiaowei Li, Guocai Zhang, Chao Ma, Xianjun Dai

**Affiliations:** College of Life Sciences, China Jiliang University, Hangzhou 310018, China; yangk@cjlu.edu.cn (K.Y.); liqiaoweicjlu@163.com (Q.L.); zhanggc1996@126.com (G.Z.); machaomedical@gmail.com (C.M.)

**Keywords:** carrageenan oligosaccharides, intestinal oxidative stress, gut flora, *Drosophila melanogaster*

## Abstract

Carrageenan oligosaccharides (COS) have been reported to possess excellent antioxidant activities, but the underlying mechanism remains poorly understood. In this study, H_2_O_2_ was applied to trigger oxidative stress. The results showed that the addition of COS could effectively extend the lifespan of female *Drosophila*, which was associated with improvements by COS on the antioxidant defense system, including a decrease in MDA, the enhanced activities of SOD and CAT, the reduction of ROS in intestinal epithelial cells, and the up-regulation of antioxidant-relevant genes (*GCL*, *GSTs*, *Nrf2*, *SOD*). Meanwhile, the axenic female *Drosophila* fed with COS showed almost no improvement in the above measurements after H_2_O_2_ treatment, which highlighted the antioxidant mechanism of COS was closely related to intestinal microorganisms. Then, 16S rDNA high-throughput sequencing was applied and the result showed that the addition of COS in diets contributed to the diversity and abundance of intestinal flora in H_2_O_2_ induced female *Drosophila*. Moreover, COS significantly inhibited the expression of gene *mTOR*, elevated its downstream gene *4E-BP*, and further inhibited autophagy-relevant genes (*AMPKα*, *Atg1*, *Atg5*, *Atg8a*) in H_2_O_2_ induced female *Drosophila*. The inhibition of the mTOR pathway and the activation of autophagy was probably mediated by the antioxidant effects of COS. These results provide potential evidence for further understanding of COS as an intestinal antioxidant.

## 1. Introduction

Oxidative stress, the unbalanced biological reactions which cause the number of free radicals to increase in the body, which in turn damage biological macromolecules such as DNA, lipids and proteins, plays an important role in the pathogenesis of many chronic-degenerative disorders [1,2]. Due to modern lifestyles, such as processed foods, exposure to various toxic species, and lack of exercise, oxidative stress damage inevitably occurs. The gut is an important source of reactive oxygen species (ROS) due to its inevitable exposure to foreign substances and microbial pathogens [3,4,5]. When external harmful substances stimulate the intestine to cause oxidative stress, the intestinal epithelial cells will act as the first barrier to create a direct immune response. A large number of ROS and antimicrobial peptides (AMPs) will be generated during this process [6]. At low or moderate amounts, ROS are beneficial for a series of cellular functions, but excessive ROS production leads to the disruption of important cellular processes [7,8]. To alleviate intestinal injury, the dynamic balance of ROS generation is a vital factor in maintaining the homeostasis of the intestinal environment. Therefore, attention is more focused on safe antioxidants ingested through the diet.

Carrageenans are the general name of the sulfated polysaccharide family, mainly derived from the cell wall of red seaweed [9]. It has been demonstrated that carrageenans could act as potential ROS scavengers to protect living organisms from oxidative damage [10]. However, the application of carrageenan is greatly restricted due to its high molecular weight. Carrageenan oligosaccharides (COS) can be prepared from carrageenan. Sun et al. compared different kinds of κ-carrageenan oligosaccharides and considered that COS prepared from H_2_O_2_ depolymerization could be used as a potential antioxidant [11]. During the preparation process, the rupture of sugar chains causes a large number of active groups to become exposed and may enhance immune regulation activities. In addition, COS also possesses multiple biological activities, including antitumor, antivirus, anti-inflammatory and anticoagulant [12]. Nevertheless, studies concerning the influences of COS on intestinal immune function are still limited.

*Drosophila melanogaster* (fruit fly) has been proved to be a powerful animal model for genetic and epigenetic research [13]. Recent evidence suggests that the intestinal-targeted expression of antioxidant enzymes or antioxidant compounds could clear ROS from the gut and then allow a normal extension of lifespan of *Drosophila* [14,15]. Gut microbes play an important role in host nutritional intake by their catabolism of nutrients or biosynthesis of metabolites [16,17,18]. Additionally, gut microbiota was proved to be important in aging and the relevant neurodegenerative disorders in *Drosophila melanogaster* [19]. For marine oligosaccharides, *Drosophila melanogaster* was used as a model to explore the anti-aging effect of agar oligosaccharide [20]. For COS, the current authors’ previous study showed its potential antioxidant capability on the lifespan extension of male *Drosophila melanogaster*, which was associated with immunity and gut microbiota [21]. It is not clear whether COS have the same effect on female flies, and little is known about how COS diets relate to microbial composition and immune response.

In view of the previous research, the protective effects of COS on the intestinal oxidative stress induced by hydrogen peroxide (H_2_O_2_) in female *Drosophila* model were evaluated in this study. COS diets and a normal diet were applied, to compare the apparent phenomena of the life span of *Drosophila* and antioxidant enzyme activities in vivo. The changes of intestinal epithelial cell death and ROS level were detected and determined using immunostaining method, and the intestinal morphology were observed through a microscope. Furthermore, gut bacterial community composition and gene expression associated with intestinal immune responses were also analyzed. In particular, axenic *Drosophila* were generated to verify whether COS play a role for the intestinal microbiome. This study aims to extend the understanding of COS antioxidant biological activity, and provide a theoretical basis for COS to alleviate intestinal damage, which is of great significance to promote the use and development of COS.

## 2. Materials and Methods

### 2.1. Materials

Pharmaceutical grade carrageenan oligosaccharides (COS, ≥95%) were obtained from Qingdao Bozhihuili Biological Technology Co., Ltd., Qingdao, China. The COS were prepared through the acid hydrolysis of carrageenan, which were mainly with degrees of depolymerization of 3–13, as determined by HPLC. Reagents 7-aminoactionomycin D (7-AAD), dihydroethidium (DHE), catalase (CAT) kit, malondialdehyde (MDA) kit, superoxide dismutase (SOD) kit, and Coomassie blue staining kit were obtained from Nanjing Jiancheng Biological Engineering Co., Ltd., Nanjing, China.

### 2.2. Fly Stocks

Canton-S wild type *Drosophila melanogaster* were acquired from the *Drosophila* Stock Center at Shanghai Academy of Life Sciences. Based on the results of previous research [21], female fruit flies were used in this study. The *Drosophila* were reared at 25 °C with a 12 h light/12 h dark cycle under 55% relative humidity. In this study, the *Drosophila* were fed with a standard medium composed of corn, yeast and agar. Preliminary in vitro experiments were performed to determine the optimal concentration for in vitro administration, which was determined according to the following formula: experimental in vitro drug concentration (µg/mL) = 50 × D/5000 ÷ 50% × 10^3^ (where D is the clinical dose mg.kg day). The optimal dose of COS for the *Drosophila* in experiment was calculated to equal 0.125%. The optimal dose of COS (0.125%) was set as the medium dose; the low-dose and high-dose groups were decreased or increased on this basis. In order to assess the effect of COS on flies, COS were supplemented in the basal media to final concentrations (*w*/*w*) of 0.0625, 0.125 and 0.25, defined as COS-L, COS-M and COS-H groups, respectively.

### 2.3. Generating Axenic Drosophila

To explore the complex interaction among gut microbiota, *Drosophila* physiological activity and oxidative stress damage, axenic *Drosophila* were generated according to the report of Schretter et al. [22], with some modification. Briefly, freshly laid eggs were harvested and washed in succession with Walch solution (1:30, *v*/*v*, 3 times/min), bleach (50%, 1 min), 70% ethanol (2 times/min) and sterile water (2 times/min). Axenic nutritive substrates were prepared by adding antibiotics, including 5 µg/mL tetracycline, 20 µg/mL rifamycin and 50 µg/mL ampicillin for at least one generation. To confirm axenic *Drosophila*, fly homogenates were plated on agar plates (MRS, LB) and checked for colony formation. Similarly, different content COS were supplemented in the basal axenic media for the following experiments and expressed as A-COS-L, A-COS-M and A-COS-H respectively.

### 2.4. Oxidative Stress Treatment and Lifespan Assay

H_2_O_2_ was used to trigger the oxidative stress in *Drosophila*. The newly emerged male and female flies were separated under anesthesia with carbon dioxide. Female *Drosophila* were collected and randomly divided into different groups. For H_2_O_2_ treatment, the female *Drosophila* were transferred into an empty culture tubes hanger for 2 h, and then a filter paper, infiltrated with different solutions (150 μL), was placed inside. The non-oxidative control group was fed with 5% *m/m* sucrose (Z-SUC). The oxidative control group (Z-H_2_O_2_) was fed with 5% sucrose (*m/m*) containing 3% H_2_O_2_ (*m/m*). The other three groups (COS-L, COS-M and COS-H) were raised with 5% sucrose (*m/m*) with 0.0625, 0.125 and 0.25 COS (*m/m*) containing 3% H_2_O_2_ (*m/m*). The number of surviving fruit flies in each culture tube was recorded every 12 h. Axenic *Drosophila* were also treated following the above procedures with sterile sucrose solution, and represented as A-Z-SUC, A-Z-H_2_O_2_, A- COS-L, A-COS-M and A-COS-H respectively. At the end of the experiment, the mean lifespan, the maximum lifespan and the half time to death of female fruit flies were calculated.

### 2.5. The Oxidation Level in Drosophila

According to the above method of oxidative stress treatment (Section 2.4), the female flies were fed for 72 h, and the filter papers were changed every 12 h. After induction, the average body weight was weighed and calculated. *Drosophila* were homogenized with normal saline in an ice bath according to the ratio of M1 (fly weight):M2 (normal saline) = 1:9, centrifuged at 4 °C (2500 g/min for 15 min). Then the supernatants were collected for the determination of the activities of SOD and CAT enzyme and the content of MDA by corresponding kits (Nanjing Jiancheng Biological Engineering Co., Ltd., Nanjing, China).

### 2.6. The Intestinal Oxidative Damage in Drosophila

#### 2.6.1. The Intestinal Morphological Changes

The female flies were fed for 72 h with oxidative stress treatments as described above (Section 2.4). After induction, 5–10 fruit flies’ intestines were dissected with a dissecting microscope, then immersed in pre-cooled PBS buffer. Next, they were fixed with 4% paraformaldehyde for 15 min. Finally, the shape, length and width of fruit flies’ intestine were observed with the microscope.

#### 2.6.2. Staining

The 7-amino-actinomycin D (7-AAD) and dihydroethidium (DHE) were applied to determine the death number and ROS level of intestinal epithelial cells. Briefly, a total of 3–5 intestines were obtained in ice-cold PBS and stained with 7-AAD (10 μg/mL) or DHE (10 μmol/L) for 30 min in the dark. Next, the intestines were washed with PBT solution three times (5 min), fixed with 4% paraformaldehyde for 30 min, and washed three times again with PBT solution. Then the samples were stained with DAPI (1 μg/mL) at room temperature in the dark for 7–8 min, washed twice with PBT solution and observed with a fluorescence microscope.

### 2.7. Analyses of the Gut Microbiota

#### DNA Extraction and 16S rDNA Sequencing

Female fruit flies were washed with 70% ethanol, and the midguts were obtained. According to the manufacturer’s instructions, DNA were extracted from different samples with E.Z.N.A. ^®^Stool DNA Kit (D4015, Omega). Primers 341F (5′-GTGYCAGCMGCCGCGGTAA-3′) and 805R (5′-GGACTACNVGGGTWTCTAA-3′) were used to amplify the V3-V4 region of the prokaryotic small subunit (16S) rRNA gene. The PCR products were confirmed with 1.5% agarose gel electrophoresis. Then they were purified by AMPure XT beads (Beckman Coulter Genomics, Massachusetts, United States) and quantified by Qubit (Invitrogen). The 16S rDNA sequencing was assessed by reference to the previous study [21,23]. Briefly, the amplicon pools were prepared for sequencing and the size and quantity of the amplicon library was assessed on an Agilent 2100 Bioanalyzer (Agilent, Palo Alto, USA) and with the Library Quantification Kit for Illumina (Kapa Biosciences, Woburn, MA, USA), respectively. The libraries were sequenced on NovaSeq PE250 platform. Illumina NovaSeq platform was used for sequencing. Alpha diversity (α-diversity) and beta diversity (β-diversity) were analyzed with QIIME2, and figures were drawn by R language (v3.5.2, R Core Team, Vienna, Austria).

### 2.8. Analysis of Gene Expression Relevant to Intestinal Oxidative Stress Damage

A total of 30 intestines were obtained and the total RNA of flies’ intestines were extracted using the RNAiso Plus Kit (9108Q, Takara Bio Inc., Dalian, China). The cDNA was constructed by using the PrimeScript™ RT reagent Kit with gDNA Eraser (RR047Q, Takara Bio Inc., Dalian, China) and the primers used in this study are shown in Table 1. The RT-qPCR was performed using TB Green^®^ Premix Ex Taq™ (RR420Q, Takara Bio Inc.) on the qTOWER3 Real-Time System (Analytik Jena, Jena, German). The PCR program was as follows: an initial temperature of 94 °C for 4 min followed by 40 thermal cycles of 94 °C for 5 s and 60 °C for 30 s. The relative mRNA expressions were analyzed by 2^−ΔΔCt^ method with the gene *RP49* as a housekeeping gene for normalization.

### 2.9. Statistical Analysis

At least 3 independent experiments were repeated in this study. GraphPad Prism 6.0 (GraphPad Software, San Diego, USA)was used for statistical analysis, and Image J (National Institutes of Health, Bethesda, USA) was applied for image analysis. Differences among treatments were obtained with one-way analysis of variance followed by Tukey’s multiple comparisons test. *p* > 0.05 indicated that there was no significant difference, * represented *p* < 0.05; ** represented *p* < 0.01.

## 3. Results and Discussion

H_2_O_2_ is a strong oxidant. Some studies have reported that H_2_O_2_ induction caused the generation of a large number of hydroxyl radicals and the acceleration of aging [15,24]. In this study, H_2_O_2_ was used to trigger oxidative stress and the survival rate of female flies (normal flies and axenic flies) were detected. The current authors’ previous study analyzed the effect of COS on the longevity and oxidative stress in male *Drosophila* [21]. Following that research, the authors tried to explore whether COS have the same effect on female flies. Interestingly, compared with male flies, the results showed that COS was more beneficial to the oxidative stress resistance of female flies, especially in extending lifespan and improving antioxidant defense system (SOD and CAT activities, and CAT content). Therefore, to better investigate the influence of gut flora related to COS antioxidant capability, the authors focused on the female of *Drosophila* in this study.

### 3.1. Effect of COS on the Lifespan in of Female Flies with Oxidative Stress

**Figure 1:**
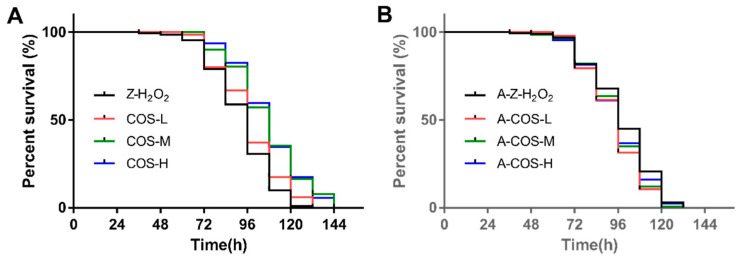
The survival curve of H_2_O_2_-induced female *Drosophila* with different feeding: (**A**) normal fruit flies, (**B**) axenic fruit flies.

**Figure 2:**
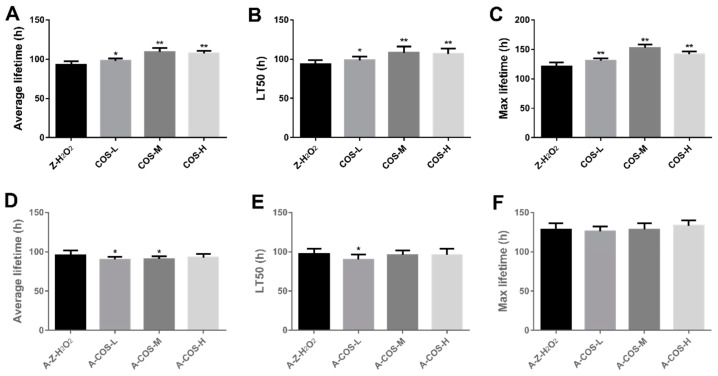
Effect of COS on the average lifespan (**A**,**D**), half time to death (**B**,**E**) and maximum lifespan (**C**,**F**) of H_2_O_2_ treated *Drosophila.* Charts (**A**–**C**) present the results of normal fruit flies, and (**D**–**F**) present the results of axenic fruit flies. Differences among treatments were obtained with one-way analysis of variance followed by Tukey’s multiple comparisons test. When compared with the H_2_O_2_ treated group, * and ** represent *p* < 0.05 and *p* < 0.01 respectively.

Since fruit flies fed with sucrose were almost all alive within 144 h, the data of its life span result were not shown. For normal female flies, compared with the control group (Z-H_2_O_2_ group), the viability of female fruit flies fed with COS was significantly improved. As shown in Figure 1A, when fruit flies were treated with H_2_O_2_ for 96 h, the survival rate of the Z-H_2_O_2_ group was reduced to 30.5%, while the survival rate of female fruit flies fed with 0.0625%, 0.125% and 0.25% COS were 37.0%, 59.5% and 59.5%, respectively. In addition, the mean lifespan, the median lifespan and the maximum lifespan were calculated as shown in Figure 2A–C. Compared with the control group (Z-H_2_O_2_ group), the mean lifespan (Figure 2A) was significantly increased in COS-fed female flies by 5.44% (*p* < 0.05), 17.8% (*p* < 0.01) and 15.7% (*p* < 0.01), and similar results were achieved for the medium lifespan and maximum lifespan (Figure 2B,C). Above all, COS showed positive effects on the protection against H_2_O_2_-induced oxidative stress in *Drosophila*.

Axenic *Drosophila* were generated to explore whether COS could prolong the life span of female *Drosophila* through the gut microbiome. As shown in Figure 1B, axenic female flies with COS diets presented a lower survival rate in comparison with the A-Z-H_2_O_2_ group. It can be seen from Figure 2D that the average lifespan of 0.0625% and 0.125% COS-fed axenic groups were slightly lower than that of the A-Z-H_2_O_2_ group. Only the 0.0625% COS-fed group presented little difference in the medium lifespan from the A-Z-H_2_O_2_ group. No significant differences were found in maximum lifespan between COS-fed groups and the axenic A-Z-H_2_O_2_ group. *Drosophila* possess complex gut structures and organization similar to those of the mammalian gut, hence fruit flies are considered to be an effective model for exploring the functional effects of substances on gut integrity relevant to aging [25]. Moreover, there is emerging evidence of the association between microbe-mediated changes and host physiology [26]. According to the results obtained in axenic *Drosophila*, it highlighted that COS worked through gut microbes then alleviate the oxidative stress effect of hydrogen peroxide to extend the lifespan of fruit flies.

### 3.2. Influences of COS on the Antioxidant Ability of Female Drosophila

**Figure 3:**
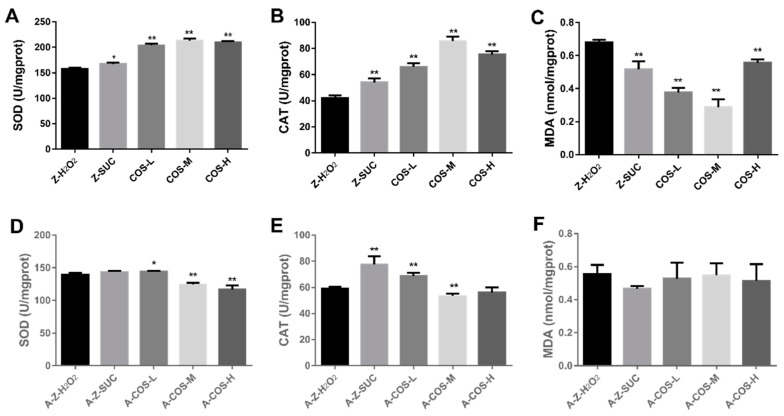
Effects of COS on SOD (**A**,**D**), CAT (**B**,**E**) and MDA (**C**,**F**) levels in female *Drosophila*. Charts (**A**–**C**) present the results of normal fruit flies, and (**D**–**F**) present the results of axenic fruit flies. Differences among treatments were obtained with one-way analysis of variance followed by Tukey’s multiple comparisons test. When compared with the H_2_O_2_ treated group, * and ** represent *p* < 0.05 and *p* < 0.01 respectively.

A variety of antioxidant enzymes are the extremely significant part of the body’s defense system, which could maintain ROS dynamic balance and prevent excessive ROS from causing oxidative damage to the body [27]. SOD can scavenge harmful free radicals to prevent damage to the body and CAT can dissolve harmful peroxides in the body into water, which all play important roles in anti-oxidation. Therefore, the effects of COS on SOD and CAT activities in oxidatively stressed flies were analyzed. As shown in Figure 3A,B, SOD and CAT activities were significantly increased with the addition of COS in the diets of normal female *Drosophila*. Compared with the Z-H_2_O_2_ group, under oxidative stress, the SOD activity of COS-fed groups increased by 29.4% (*p* < 0.01), 35.2% (*p* < 0.01) and 33.4% (*p* < 0.01), and the CAT activity of COS-fed groups increased by 55.3% (*p* < 0.01), 102% (*p* < 0.01) and 80.0% (*p* < 0.01). Additionally, MDA levels of Z-H_2_O_2_, Z-SUC, COS-L, COS-M and COS-H were also assessed because MDA is an indicator of lipid peroxidation and oxidative damage, which is cytotoxic and causes body damage [28]. As can be seen from Figure 3C, compared with Z-SUC, H_2_O_2_ induction could significantly increase the MDA content in female fruit flies, indicating that the induction of H_2_O_2_ caused the formation of lipid peroxides. Different doses of COS could effectively reduce the MDA content in fruit flies by 44.5% (*p* < 0.01, COS-L), 57.7% (*p* < 0.01, COS-M) and 17.8% (*p* < 0.01, COS-H) respectively.

The effects of COS on antioxidant abilities in axenic female flies were detected as shown in Figure 3D–F. It showed that SOD enzyme activity increased in the low concentration group, but decreased in the medium concentration and high concentration groups. Compared with the A-Z-H_2_O_2_ group, the CAT activity of A-Z-SUC and A-COS-L groups were increased by 30.9% (*p* < 0.01) and 16.1% (*p* < 0.01), and A-COS-M group was reduced by 9.99% (*p* < 0.01). No statistically significant difference was noticed in MDA content between A-Z-H_2_O_2_ and COS-treated groups. Taking the above results into consideration, it suggested that COS could not effectively increase the SOD and CAT activities and reduce the MDA content in axenic fruit flies under oxidative stress conditions. This result may be explained by previous studies where COS increased inflammatory effects, maintaining secretion of pro-inflammatory cytokines, while SCFAs, which could suppress the production of pro-inflammatory cytokines, were the main fermentation products of indigestible carbohydrates metabolized by intestinal bacteria [29,30,31]. Therefore, without the utilization and protection of gut microbiota, it might not be possible for COS to protect axenic female *Drosophila* from oxidative stress.

### 3.3. The Effect of COS on Intestinal Oxidative Damage in Drosophila

#### 3.3.1. The Changes of Intestinal Integrity

**Figure 4:**
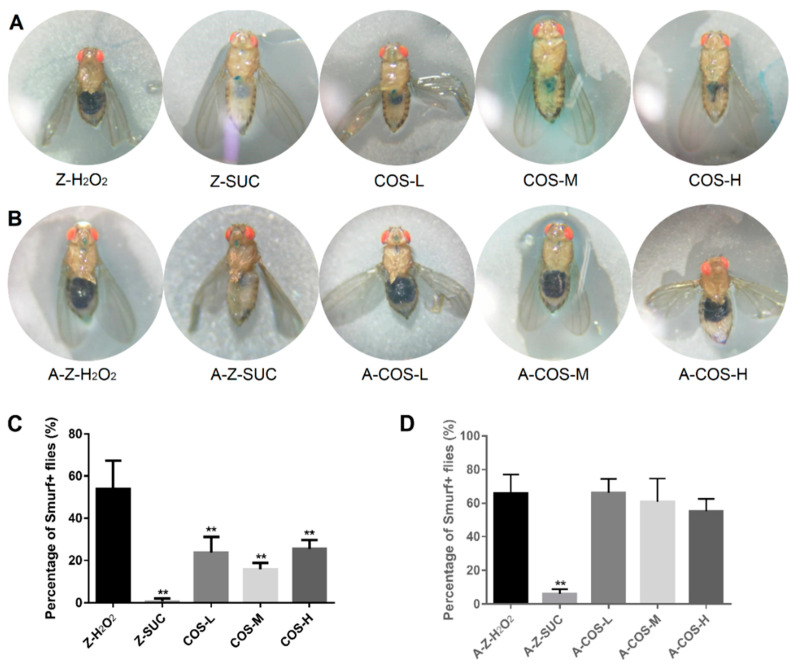
The effect of COS on gut integrity in female *Drosophila*. The dye of FD & C Blue No.1 is displayed in female *Drosophila* (**A**) and axenic female *Drosophila* (**B**) with different treatments. The percentage of “Smurf” flies in normal and axenic types are shown in (**C**) and (**D**) respectively. Differences among treatments were obtained with one-way analysis of variance followed by Tukey’s multiple comparisons test. When compared with the H_2_O_2_ treated group, ** represent *p* < 0.01 respectively.

Fruit flies possess good intestinal function and intestinal integrity, but the intestinal homeostasis will be disrupted under oxidative stress conditions, leading to impaired intestinal barrier function. Therefore, the intestinal leak was monitored with Smurf assay in this study. If the dye only showed from the mouthparts to the digestive tract, it was considered that the intestines were not leaking (such as Figure 4 Z-SUC). Otherwise, when the dye occurred in the abdominal cavity, head, chest and other parts, it was judged to be intestinal barrier dysfunction, such as Figure 4 Z-H_2_O_2_. As shown in Figure 4A, intestinal barrier dysfunction occurred in H_2_O_2_ treated normal female fruit flies, while the intestines of *Drosophila* in the Z-SUC group were intact without leakage. After being fed with COS, their intestinal integrity was better and there was no leakage in comparison with the Z-H_2_O_2_ group. Additionally, the percent of Smurf+ flies (Figure 4C) were analyzed to further confirm the above results. The Z-SUC group showed almost no intestine leakage. Compared with the Z-H_2_O_2_ group, the percent of Smurf+ flies in COS feeding groups were decreased by 30.28% (COS-L, *p* < 0.01), 38.22% (COS-M, *p* < 0.01) and 28.54% (COS-H, *p* < 0.01). These results indicated that COS have the effect of protecting the integrity of the intestinal tract of female fruit flies.

In order to investigate whether the improvement of intestinal integrity by COS still occurred in an axenic background, Smurf assays were performed using axenic female *Drosophila* under aseptic conditions. As shown in Figure 4B,D, supplementation of different content of COS could hardly decrease the percent of Smurf+ flies with oxidative damage. Hiroki Nagai and Tamaki Yano [32] found that autophagy in intestinal cells inhibited the regenerative response triggered by ROS secreted by host epithelial cells to intestinal symbiotic bacteria. In the absence of this inhibition, excessive regeneration would be caused, leading to age-dependent barrier dysfunction and systemic inflammation. In this study, there were no intestinal symbiotic bacteria in axenic female *Drosophila*, so suppression of the regenerative response might not have been triggered. Based on these data, it is suggested that COS might improve intestinal integrity through the modulation of gut microbiota-related autophagy pathways.

#### 3.3.2. The Changes of Drosophila Gut Morphology

**Figure 5:**
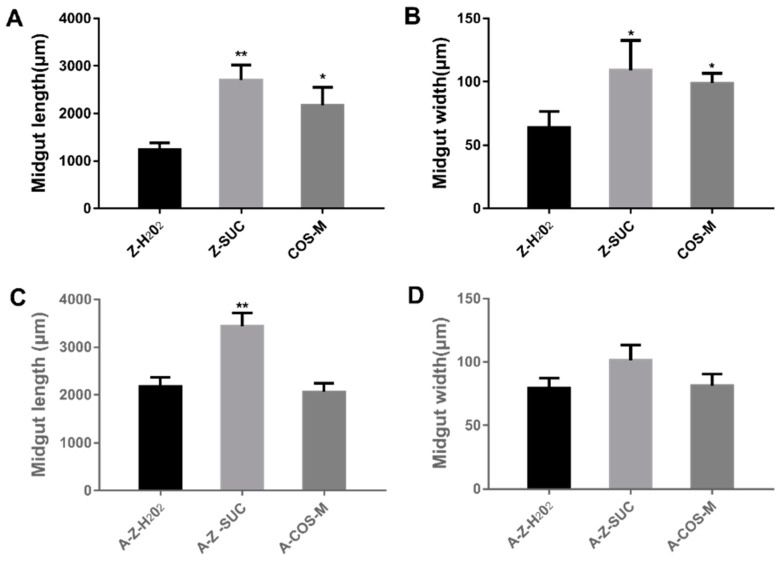
The length and width of guts in female fruit flies (**A**,**B**) and axenic fruit flies (**C**,**D**). Differences among treatments were obtained with one-way analysis of variance followed by Tukey’s multiple comparisons test. When compared with the H_2_O_2_ treated group, * and ** represent *p* < 0.05 and *p* < 0.01 respectively.

Oxidative stress may cause changes in the gut morphology of fruit flies. In this study, the *Drosophila* midgut was isolated and observed using a microscope after 72 h of H_2_O_2_ induction. As 0.125% COS fed groups showed better antioxidant effect and longer lifespan, the following experiments were carried out with Z-H_2_O_2_, Z-SUC and COS-M groups. Figure 5 showed that the length and the width of midguts in female fruit flies fed with sucrose were healthy and normal, while that of female fruit flies treated with H_2_O_2_ were significantly decreased. Compared with axenic flies, COS could maintain gut morphology with the gut microbiota. Throughout an animal’s life, mature organs can undergo continuous cell renewal but remain roughly the same size [33]. The timely renewal of intestinal epidermal cells is necessary for the repair of intestinal injury. In order to maintain intestinal homeostasis, dead cells must be replaced by new ones differentiated from intestinal stem cells [34,35]. Compared with the flies fed with sucrose, the intestines of flies induced by hydrogen oxide became shorter and narrower, indicating that there may be more intestinal necrotic cells, and that the differentiation of intestinal stem cells was reduced. COS supplementation contributed to the maintenance of intestinal morphology (Figure 5A,B), suggesting that COS played a role in maintaining intestinal homeostasis after injury. At the same time, the results of axenic fruit flies indicated that COS worked in maintaining intestinal homeostasis with the indispensable role of gut microbes.

### 3.4. Effects of COS on the Changes of Intestinal Cell Activity and ROS Levels in Drosophila

**Figure 6:**
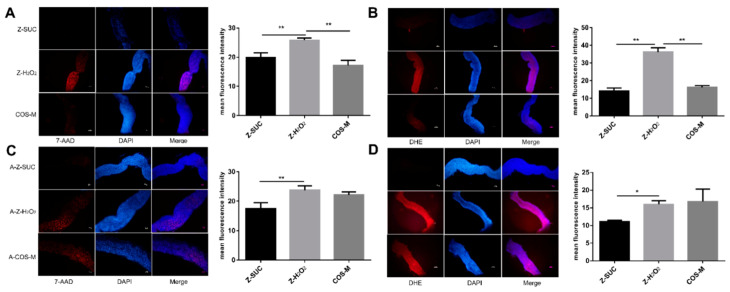
Gut dissected from female *Drosophila* (**A**,**C**) or axenic female *Drosophila* (**B**,**D**) with different treatments were stained with 7-AAD (red) and DHE (red) respectively. Nuclei were counterstained with DAPI (blue). Scale bar: 50 μm. Differences among treatments were obtained with one-way analysis of variance followed by Tukey’s multiple comparisons test, * and ** represent *p* < 0.05 and *p* < 0.01 respectively.

To further investigate whether the change of gut morphology resulted from cell death after the induction of H_2_O_2_, dissected guts which had been exposed to H_2_O_2_ for 72 h were stained with 7-AAD. Additionally, the reduced survival rate of *Drosophila* under oxidative stress may be associated with the destruction of ROS dynamic balance in the intestine of *Drosophila*. Therefore, DHE was applied to determine the ROS level in the gut. It can be seen from Figure 6A that *Drosophila* fed with 5% sucrose showed a very small number of death intestinal epithelial cells. After induction by hydrogen peroxide, a large number of intestinal epithelial cells died. Compared with the Z-H_2_O_2_ group, COS could significantly decrease the death number of *Drosophila* intestinal epithelial cells. Similarly, the ROS level in the Z-SUC group was low, while in the Z-H_2_O_2_ group it was high. The ROS level in COS-fed *Drosophila* indicated the effective antioxidant activity of COS. Taken together, these results suggested that COS had a positive effect on eliminating excess ROS generated by H_2_O_2_ oxidative stress, thereby alleviating oxidative stress damage and maintaining intestinal homeostasis.

Likewise, axenic *Drosophila* were also used to explore the role of gut microbe in the gut cell death and gut ROS level as shown in Figure 6B. In the A-Z-SUC group, the death number of gut epithelial cell was small, and the ROS level in the intestine was very low. Under oxidative stress, the intestinal epithelial cells in axenic female *Drosophila* died significantly and produced a large amount of ROS. After feeding COS, the number of intestinal epithelial cell deaths and ROS levels of axenic *Drosophila* showed not much change compared with the A-Z-H_2_O_2_ group. Similarly, Wikoff et al. [36] found that the processing of indole-containing molecules was enhanced by the gut microbiome in germfree mice. Moreover, Poeggeler et al. [37] used an in-vitro aging model that showed that an indole derivative was helpful in stabilizing energy metabolism and reducing ROS production. Therefore, it could be inferred that the damage caused by oxidative stress could be removed by COS with the help of the action of intestinal microorganisms.

### 3.5. The Effects of COS on Intestinal Microbiota of Female Drosophila

**Figure 7:**
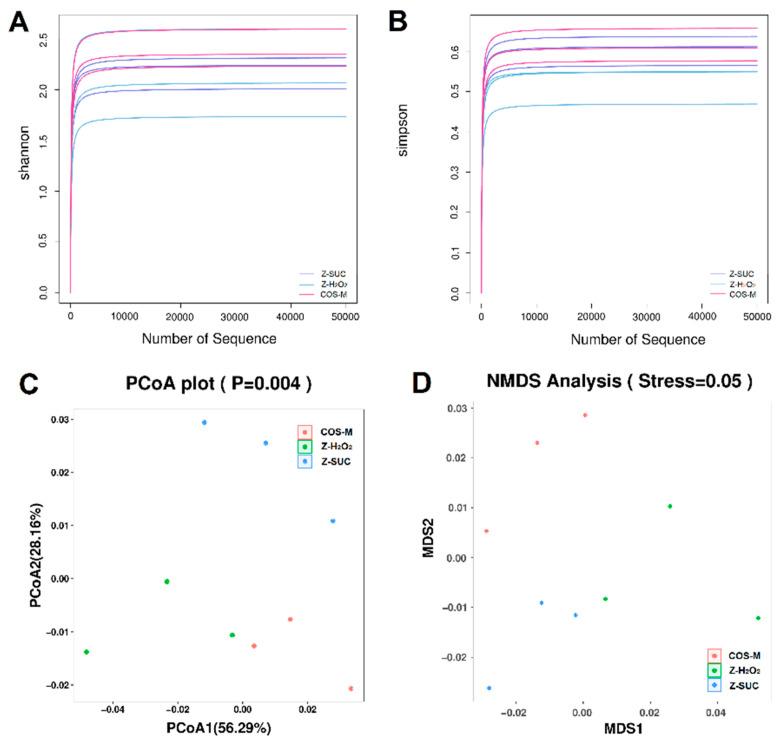
Analysis of alpha diversity and beta diversity of the intestinal microbiota: (**A**) Shannon index, (**B**) Simpson index, (**C**) principal co-ordinate analysis (PCoA), and (**D**) nonmetric multidimensional scaling (NMDS) analysis.

In view of the above results of axenic female *Drosophila*, 16S rDNA sequencing was used to analyze the microbial composition of female *Drosophila* of the Z-SUC, Z-H_2_O_2_ and COS-M groups. Firstly, the α-diversity (Figure 7A) was analyzed to reflect the diversity of the gut microbial system in female *Drosophila* with different treatments. As shown in Figure 7B, principal co-ordinate analysis (PCoA) and nonmetric multidimensional scaling (NMDS) were used to present the β-diversity analysis. The results of PCoA (Figure 7C) showed that PCoA1 and PCoA2 accounted for 56.29% and 28.16% of the overall difference in the flora structure, respectively, and there were clear distinctions in the gut microbiota community structure among Z-H_2_O_2_, Z-SUC and COS-M groups. Similarly, the result of NMDS (Figure 7D) yielded the same results.

**Figure 8:**
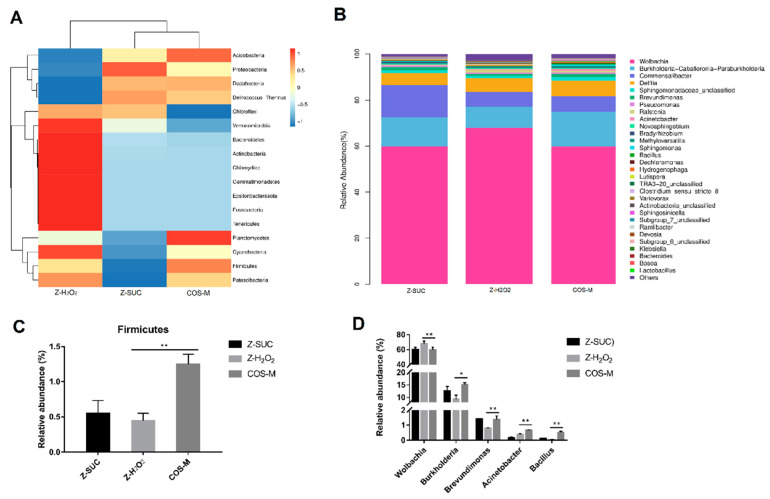
The relative abundance of the gut flora of Z-H_2_O_2_, Z-SUC, and COS-M groups at the phylum level (**A**), and genus level (**B**). The relative abundance of the gut flora of Z-H_2_O_2_, Z-SUC, and COS-M groups with significant differences at the phylum level (**C**), and genus level (**D**). Differences among treatments were obtained with one-way analysis of variance followed by Tukey’s multiple comparisons test, * and ** represent *p* < 0.05 and *p* < 0.01 respectively.

The effect of COS intake on the abundance of flora was also analyzed at the phylum level (Figure 8A,C). In comparison to Z-H_2_O_2_ group, the intake of COS could significantly increase the abundance of *Firmicutes* from 0.45% to 1.25% (*p* < 0.01). In accordance with the present result, Niu et al. [38] found that the relative abundances of *Firmicutes* increased significantly during the co-composting of bagasse pith and dairy manure in the presence of H_2_O_2_. A previous study showed that *Firmicutes* was important in carbohydrates utilization and cellulose degradation [39]. This indicated that more carbohydrates such as COS might be utilized rapidly by *Firmicutes*. At the level of genus classification (Figure 8B,D), *Wolbachia*, *Burkholderia*, *Commensalibacter*, and *Delftia* were dominant in this system. Compared with the Z-H_2_O_2_ group, it was highlighted that the intake of COS significantly decreased the abundance of Wolbachia from 67.94% to 59.80% (*p* < 0.01), while it increased the abundance of *Burkholderia*, *Brevundimonas*, *Acinetobacter* and *Bacillus* from 9.22%, 0.79%, 0.38% and 0.05%, to 15.09%, 1.38%, 0.86% and 0.68%, respectively. Though *endosymbiotic Wolbachia* bacteria was shown to influence various aspects of insect biology, the presence of *Wolbachia* bacteria did not cause significant changes in the Imd and ROS pathways [40], which are closely related to intestinal immunity and anti-oxidation. Notably, the relative abundance of *Burkholderia* increased significantly with the treatment of COS. *Burkholderia* gut symbionts are known to enhance the innate immunity in *Riptortus pedestris* [41]. Therefore, it seems that *Burkholderia* played an important role in the gut from oxidative stress.

**Figure 9:**
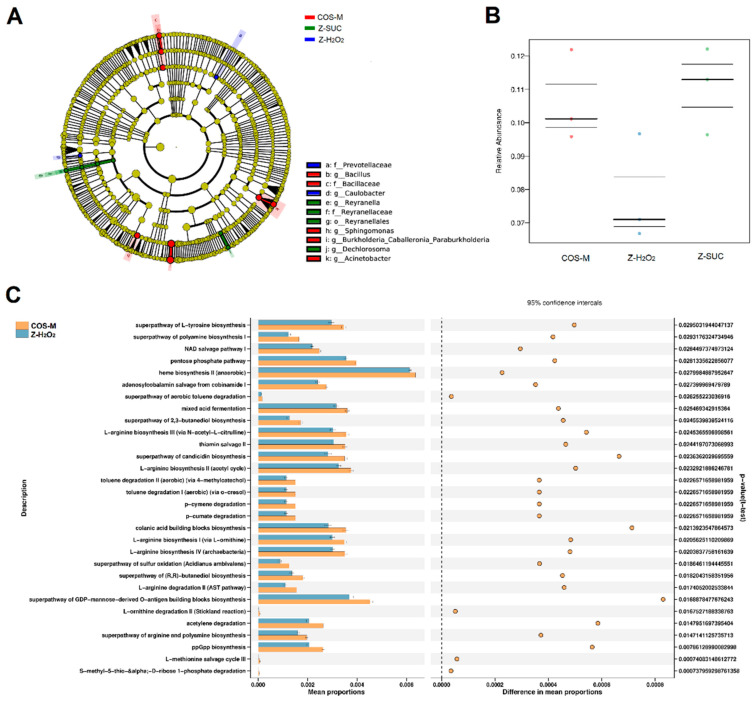
Carrageenan oligosaccharides alter the gut microbiota and the relevant pathways of H_2_O_2_-induced female *Drosophila*. (**A**) The cladogram of the gut microbiota community of female *Drosophila* in Z-H_2_O_2_, Z-SUC and COS-M groups. (**B**) Prediction of the relative abundance of stress-tolerant bacteria of the gut microbiota of female *Drosophila* in Z-H_2_O_2_, Z-SUC and COS-M groups using BugBase. (**C**) KEGG pathways predicted using PICRUSt 2, and the t-test was used to perform a significance analysis.

In order to further explore the community differences in Z-H_2_O_2_, Z-SUC and COS-M groups, LEfSe analysis of the intestinal flora of female *Drosophila* was carried out as shown in Figure 9A. The results presented the dominant genera of Z-SUC group were *Reyranella* and *Dechlorosoma*, the dominant genera of the Z-H_2_O_2_ group was *Caulobacter*, and the dominant genera of COS-M group were *Bacillus*, *Sphingomonas*, *Burkholderia* and *Acinetobacter*. It can be seen that the intake of COS significantly increased the diversity of the gut flora. At the same time, the microbial communities in the host were involved in many aspects of body health, including metabolism, immune response, disease status, and even behavior. Therefore, the microbiome phenotypes played an important role in understanding the potential functional capabilities of microorganisms in the gut flora. As can be seen from the Figure 9B, the intake of COS could increase the relative abundance of stress-resistant bacteria to a certain extent, while the relative abundance of stress-resistant bacteria in the gut of Z-H_2_O_2_ group decreased. These results showed that H_2_O_2_ caused the oxidative stress, leading to the death of stress-resistant bacteria, and COS was useful for the stress-resistant bacteria against oxidative stress. Additionally, PICRUSt 2 was used to predict the enriched KEGG pathways of microorganisms in Z-H_2_O_2_ and COS-M groups. As shown in Figure 9C, the proportions of pathways related to carbohydrate metabolism, amino acid metabolism, organic acid metabolism and butanediol biosynthesis increased significantly in the COS-M group in comparison with the Z-H_2_O_2_ group (*p* < 0.05). It is apparent from Figure 9C that some pathways were associated with arginine metabolism and polyamine biosynthesis. Agmatine produced by the catabolism of arginine was considered to be part of the polyamine synthesis pathway, and previous studies suggested that agmatine might be anti-inflammatory through the inhibition of nitric oxide synthase [42,43]. The most interesting thing shown in Figure 9C is that COS contributed to the pathway of colanic acid building blocks biosynthesis. Han et al. discovered that the increased secretion of the polysaccharide colanic acid promoted *Caenorhabditis elegans* lifespan by regulating mitochondrial dynamics and unfolding protein response [44]. Moreover, the proportion of butanediol biosynthesis pathway was also found to be increased in the COS-M group. The end products of butanediol could be the short-chain fatty acids as described previously, which are mainly involved in intestinal energy supply, and will affect the pH of the intestinal lumen, the permeability of the intestinal mucosal barrier, and can also regulate immunity and anti-tumor effects [42]. These results further confirmed the association between gut microbiota and their protective effects on oxidative damage.

### 3.6. Gene Expression Level of Female Drosophila Related to Immune Defense

**Figure 10:**
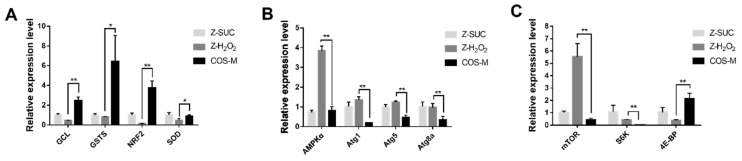
The relative expression levels of target genes in Z-SUC, Z-H_2_O_2_ and COS-M groups. (**A**) Genes associated with antioxidant activities. (**B**) Genes relevant to autophagy. (**C**) Immune-related genes. Differences among treatments were obtained with one-way analysis of variance followed by Tukey’s multiple comparisons test. * and ** represent *p* < 0.05 and *p* < 0.01 respectively.

To further explore the antioxidant mechanism of COS, RT-qPCR was used to analyze the relevant gene expression level. Four genes (*GCL*, *GSTS*, *NRF2*, *SOD*) associated with antioxidant activities were selected and detected as shown in Figure 10A. The non-oxidative control group (Z-SUC) was used to determine the relative expression level of genes in the Z-H_2_O_2_ and COS-M groups. Oxidative stress resulted in the decreased expression of the four antioxidant genes in flies, but they were significantly up-regulated after feeding COS. Compared with the Z-H_2_O_2_ group, the expression level of *GCL*, *GSTs*, *NRF2* and *SOD* in the COS-M group were extremely significantly up-regulated by a 5.66-fold change (*p* < 0.01), 7.94-fold change (*p* < 0.05), 28.5-fold change (*p* < 0.01) and 1.91-fold change (*p* < 0.05). Under oxidative stress, GCL (glutamyl-cysteine ligase) and GSTs (glutathione S-transferase) are involved in the synthesis of glutathione, which are central to redox homeostasis and redox signaling [45,46]. As for NRF2, it was reported that H_2_O_2_-induced adaptation to oxidative stress is strongly dependent on an NRF2 transcription factor-mediated increase in the 20S proteasome [47,48]. Consistent with the result of SOD level antioxidant ability of female *Drosophila* in this study, the *SOD* gene also showed an up-regulation level in the COS-M group. All these results indicated the productive effects of COS on the antioxidant abilities of female flies.

Oxidative damage caused by H_2_O_2_ could result in the accumulation of damaged molecules and cells, leading to functional disorders. Autophagy can degrade damaged or loss-of-function organelles or proteins, and maintain cell renewal and homeostasis [49]. Therefore, the effect of COS on the expression level of the autophagy gene was analyzed and shown in Figure 10B. With H_2_O_2_ treatment, the *AMPKα* gene was significantly up-regulated in female flies of the Z-H_2_O_2_ group in comparison with the Z-SUC group. It was indicated that autophagy might be triggered to deal with the oxidative damage. Compared with the Z-H_2_O_2_ group, *AMPKα*, *Atg1*, *Atg5* and *Atg8a* were down-regulated by 4.71 times (*p* < 0.01), 6.81 times (*p* < 0.01), 2.60 times (*p* < 0.01) and 2.89 times (*p* < 0.01), respectively. These results suggested that the excessive autophagy might be prevented to maintain the body’s balance of clearance, after feeding COS.

The effect of COS on the expression level of immune-relevant genes was shown in Figure 10C. Compared with the Z-H_2_O_2_ group, *mTOR* and *S6K* were significantly down-regulated in COS-M group by 32.4 times (*p* < 0.01) and 12.8 times (*p* < 0.01), respectively, while *4E-BP* was significantly up-regulated by 5.70 times (*p* < 0.01). A previous study reported that the lifespan of fruit flies and mammals could be extended after the inhibition of the mTOR pathway [50]. The mTOR kinase is considered to be the core protein of the mTOR pathway, and its downstream effectors S6K and 4E-BP regulate the transcription and the growth of fruit flies [51]. In this study, the upstream gene *mTOR* was significantly down-regulated in COS-feeding flies, whereas the downstream gene *4E-BP* was markedly up-regulated, suggesting the inhibition of the mTOR pathway after COS intake in H_2_O_2_-induced female flies. The results were similar to the study of Han et al., which suggested that purple sweet potato extract inhibited the mTOR pathway and further extended the lifespan of male *Drosophila* [52].

### 3.7. Future Research Directions

Despite progress made, there are limitations on the current work performed on the *Drosophila melanogaster* model. Therefore, to explore the application of COS in real product development or clinical utilization, further studies (such as mammalian testing) are needed to uncover the proper concentration of COS supplementation and the involved mechanism. Although the main point of this research was to determine whether COS as probiotics regulate the oxidative stress resistance of flies through the gut microbial pathway, further exploration of the molecular mechanism, including genes and proteins related to this process, is needed. To solve this problem, RT-qPCR was used to select the target gene associated with the oxidative stress resistance in this study, and the establishment of the target gene mutant combined with proteomics or transcriptomics might be helpful for further in-depth understanding of the involved mechanisms.

## 4. Conclusions

In summary, under oxidative stress conditions, COS intake could improve the survival rate of fruit flies, and effectively increase antioxidant enzyme activity in oxidatively damaged fruit flies. COS contributed to reducing the death of intestinal epithelial cells and reducing ROS in intestinal epithelial cells to alleviate intestinal oxidative damage, and maintain intestinal morphology and intestinal integrity. Further studies with axenic *Drosophila* revealed that COS must be mediated by intestinal microbes to exert their antioxidant effect. Moreover, 16S rDNA sequencing showed the intake of COS promoted the growth of beneficial bacteria to some extent, and maintained the diversity and abundance of intestinal flora. Promoting antioxidant activities, inhibiting autophagy to prevent excessive autophagy, and inhibiting the mTOR pathway to prolong lifespan may be the internal mechanism for COS to effectively alleviate the intestinal damage caused by oxidative stress. All these results are of significance in understanding the degradation or metabolism mechanism of intestinal bacteria to COS, which are food additives, and for improving its application.

## Figures and Tables

**Table 1:** Primers used in this study.

Genes	Forward Primer (5′-3′)	Reverse Primer (5′-3′)	Gene Accession Numbers
*RP49*	AGGGTATCGACAACAGAGTG	CACCAGGAACTTCTTGAATC	NM_079843.4
*AMPKα*	AGAGGTCTGCACCAAGTTCG	GTTTATTTGGTTGGCCGCGT	NM_057965.4
*Atg1*	AAGGGCAGACAAGAGTCCAT	GTTCTCCCGCTTCCTCCTTT	NM_140344.3
*Atg5*	ATATGCTTCCAGGCGGATCG	AACCACACAGCTCCATCCTG	NM_132162.4
*Atg8a*	TCTAGCCACAGCAGTTAGCG	TTGTGTAGAGTGACCGTGCG	NM_167245.3
*GCL*	GACACCGATACGCATTGCAC	CTCACCACGGAATCCTGCTT	NM_001298073.1
*GSTs*	CAGACCGTCAAGGACAACGA	TCGCGCTTGACCATGTAGTT	NM_166216.2
*Nrf2*	AGCTTCTGTCGCATGGTTGA	AGCCGTTGCTAACATGTCCA	NM_170055.2
*SOD*	ACCGACTCCAAGATTACGCTCT	GTTGCCCGTTGACTTGCTC	NM_057387.5
*mTOR*	AAAGAGCCAGACAGACGTGG	CGACGCGAAGAGTTAAAGCG	NM_057719.4
*S6K*	CGCAGGACGAGATGATGGA	TGGGATGGGTTGGTTGGT	NM_079217.3
*4E-BP*	ACCCTCTACTCCACCACTCC	GGAGTTTGGCTCAATGGGGA	NM_057947.4

## Data Availability

Data is contained within the article.
